# The Potential Role of Viral Persistence in the Post-Acute Sequelae of SARS-CoV-2 Infection (PASC)

**DOI:** 10.3390/pathogens13050388

**Published:** 2024-05-08

**Authors:** Lorenzo Lupi, Adriana Vitiello, Cristina Parolin, Arianna Calistri, Alfredo Garzino-Demo

**Affiliations:** 1 Department of Molecular Medicine, University of Padova, 35121 Padova, Italy; lorenzo.lupi.1@phd.unipd.it (L.L.); adriana.vitiello@unipd.it (A.V.); cristina.parolin@unipd.it (C.P.); arianna.calistri@unipd.it (A.C.); 2 Department of Microbial Pathogenesis, School of Dentistry, University of Maryland, Baltimore, MD 21201, USA; 3 Department of Microbiology and Immunology, School of Medicine, University of Maryland, Baltimore, MD 21201, USA

**Keywords:** post-acute sequelae of SARS-CoV-2, SARS-CoV-2 persistence, dysbiosis, PASC

## Abstract

The infection by severe acute respiratory syndrome coronavirus 2 (SARS-CoV-2) is associated not only with the development of acute disease but also with long-term symptoms or post-acute sequelae of SARS-CoV-2 (PASC). Multiple lines of evidence support that some viral antigens and RNA can persist for up to 15 months in multiple organs in the body, often after apparent clearance from the upper respiratory system, possibly leading to the persistence of symptoms. Activation of the immune system to viral antigens is observed for a prolonged time, providing indirect evidence of the persistence of viral elements after acute infection. In the gastrointestinal tract, the persistence of some antigens could stimulate the immune system, shaping the local microbiota with potential systemic effects. All of these interactions need to be investigated, taking into account predisposing factors, multiplicity of pathogenic mechanisms, and stratifying populations of vulnerable individuals, particularly women, children, and immunocompromised individuals, where SARS-CoV-2 may present additional challenges.

## 1. Post-Acute Sequelae of SARS-CoV-2

In late 2019, a novel coronavirus, subsequently named severe acute respiratory syndrome coronavirus 2 (SARS-CoV-2), was identified in patients suffering from severe pneumonia and set the beginning of the COVID-19 pandemic [1,2]. Individuals infected by SARS-CoV-2 can present a wide range of manifestations, from flu-like symptoms, such as fever and cough, to severe acute respiratory disease, characterized by a cytokine storm, vascular damage, and dissemination of intravascular coagulates [3]. Since the onset of the pandemic, it became evident that COVID-19 was not only an acute viral infection causing a short-term illness, but it can cause significant sequelae, even after weeks from the recovery from the acute phase [4]. Long-term symptoms of COVID-19 have been defined with various names, including “long COVID-19”, “post-acute sequelae of SARS-CoV-2 infection (PASC)”, “chronic COVID-19”, or “long haul COVID-19” [5]. Recently, the World Health Organization (WHO) suggested a definition of “post COVID-19” for conditions occurring in subjects 3 months after a probable or confirmed infection by SARS-CoV-2, with some symptoms persisting after at least 2 months and not attributable to other diseases [6]. In this article, we will adopt the definition of PASC as “ongoing, relapsing, or new symptoms or conditions present 30 or more days after infection”, which is consistent with the nature of the disease and has been supported by a large group of experts in the field [7]. The most common manifestations of this phase of the disease are fatigue, headache, and attention disorders [8]. Still, the symptomatology of PASC is broad and can include smell and taste disorders, cough, joint pain, impaired concentration, and memory loss, leading to a reduction of the quality of life [8,9].

While COVID-19 is considered eminently a respiratory disease, the acute phase neurological, cardiac, liver, and kidney injuries can persist [10,11]. Several pre-existing conditions are risk factors for the development of PASC, including depression, anxiety, chronic kidney disease, chronic obstructive pulmonary disease, asthma, diabetes, and autoimmune diseases, and have been analyzed and discussed elsewhere [12,13,14]. PASC could result from multiple factors, including long-term tissue damage generated during the acute phase of the disease [10,14]. Long-term symptoms occurred even in subjects with an improvement of pulmonary radiological and functional examination and completely asymptomatic patients [10,11]. Damage to brainstem function has been proposed and can be permanent, given that neurons generally cannot regenerate [10,11]. The Pretorius group discovered that fibrinogen in the blood could clot into an anomalous form of fibrin, named fibrinaloid, which is more stable and durable in blood vessels than fibrin itself, persisting for a prolonged time [15]. The formation of fibrinaloids can be induced by lipopolysaccharide (LPS) or iron ions, estrogens, lipoteichoic acid, and amyloid A [15]. Recently, the same group reported that the addition of the S1 subunit of the spike (S) protein to coagulation-competent plasma is sufficient to induce the formation of amyloids, and they suggested that the formation of these clots could lead to vascular damage and local strokes blocking the bloodstream, followed by oxygen deprivation in some areas, worsening the acute phase and possibly promoting PASC [15]. Pertinently, COVID-19, particularly in moderate and severe disease, is associated with thrombosis and thrombolytic events, mainly mediated by neutrophils and monocytes [16]. Dysregulation of the coagulation system has been associated in a transcriptomic analysis with disruption of the blood–brain barrier in individuals with neurological symptoms of PASC, including brain fog and cognitive impairment, in association with systemic inflammation [17]. In addition, it has been observed that even a mild respiratory infection of SARS-CoV-2 can induce a persistent inflammatory response in the brain, supported by the CCL11 cytokine, leading to prolonged activation of microglia in subcortical and hippocampal regions, impairing neurogenesis and myelination [18]. The effects of CCL11 are also observed after infection by Influenza virus, but unlike in SARS-CoV-2 infection, its effects are only transient [18]. PASC is associated with prolonged inflammation and lymphopenia [10,19], and it has been proposed that T cell dysfunction could cause PASC in a way similar to an auto-immune disease [20]. Relevantly, some autoantibodies can be produced during the acute phase of COVID-19 [19,21]. The infection of SARS-CoV-2, even in asymptomatic cases, can induce reactivation of latent herpesviruses (EBV, HHV6) [22,23] and endogenous retrovirus (HERV-K) [22]. This chronic viral reactivation could lead immune cells to a senescence state, prone to an aberrant and pro-inflammatory immune response [22] or directly leading to tissue damage.

In the earlier phases of the pandemic, the risk of developing PASC symptoms spanned from 50% to 80% of people who recovered from COVID-19, even in younger patients [9,24]. Recent analyses found a risk of PASC of about 43%, with an increased likelihood in patients who required hospitalization compared to people who did not require it (54% and 34%, respectively) [5]. Interestingly, infection with the Omicron variant, which is overall less virulent than the previous Delta variant, resulted in a lower risk of PASC [25,26]. To explore if SARS-CoV-2, or viral components, can persist in the body for prolonged periods, potentially causing PASC, one can directly search for its proteins or nucleic acids or examine the evolution of the specific immune response against the virus over time. Below, we review the existing data for both lines of evidence and critically summarize the potential consequences of immunopathology of PASC.

## 2. Evidence of Persistence of SARS-CoV-2

### 2.1. Persistence of Viral Nucleic Acids and Antigens

**Figure 1:**
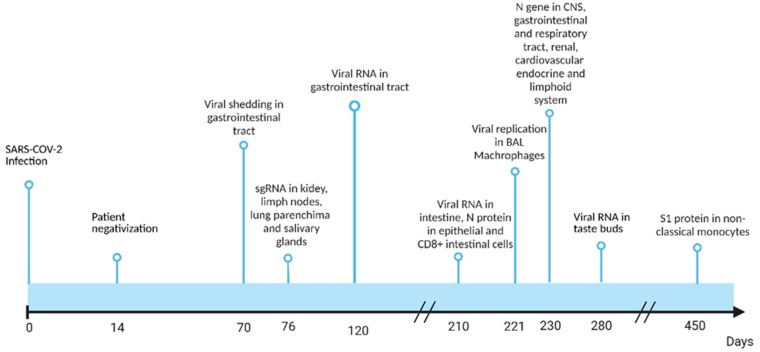
Timeline of SARS-CoV-2 nucleic acids or antigen detection after the acute phase of the disease. References are as follows: day 14 [24], day 70 [25], day 76 [30], day 120 [28], day 210 [28], day 221 [33], day 230 [30], day 280 [31], day 450 [34]. Created with BioRender.com.

In general, PASC is associated with the length of duration of viral shedding [27,28], and, overall, its incidence is reduced by antiviral therapy [29,30,31], although one study did not observe the efficacy of therapy in a cohort of cancer patients [32]. While SARS-CoV-2 is relatively rapidly removed from the upper respiratory airway, with a median time for positive-to-negative conversion of 14 days, it has been shown that SARS-CoV-2 can persist in other anatomic districts, such as the gastrointestinal tract (GIT) for a median of 19 days [33]. In a study of 69 infected children (age range: 36 h–15 years; median age: 6 years), 41 were tested by PCR both in the respiratory and gastrointestinal system (stool or anal/rectal) with a duration of viral shedding in the GIT ranging from 5 days to 5 weeks, with a mean duration of 23.6 ± 8.8 days; one child showed viral shedding even after 70 days from the disease onset (Figure 1) [34]. Among the 41 children tested both in the respiratory and gastrointestinal systems, 31 were positive for viral nucleic acid testing on their stool, rectal, or anal swabs after viral clearance from the respiratory tract. A similar pattern of delayed viral clearance from GIT was observed in a meta-analysis of several studies [34,35]. The detection of viral RNA from anal swabs was positively associated with the development of gastrointestinal symptoms and the necessity of intensive care treatment [36], highlighting the role of GIT in SARS-CoV-2 disease and possibly in PASC and viral or antigen persistence. Natarajan and colleagues studied the dynamics of viral replication in the GIT over time, between 3 to 300 days [37]. In particular, 49% of their subjects show viral RNA in fecal samples at the time of enrollment. In that same study, 78% of the patients who had gastrointestinal symptoms also manifested non-gastrointestinal symptoms, such as myalgias, decreased smell, and headache, confirming a relationship between nucleic acids (NA) detection in the GIT and long-term symptoms [37]. The proportion of patients with viral RNA in the Natarajan study gradually declined to 40% at day 28, 12.7% at day 120, and 3.8% at day 210; 0.7% of the patients remained positive for sub-genomic RNA (sgRNA) until day 120, suggesting an active replication of SARS-CoV-2 (Figure 1) [37]. Another group performed an endoscopy analysis of 46 patients affected by inflammatory bowel disease up to 291 days after infection by SARS-CoV-2 [38]. The presence of viral protein or nucleic acid was assessed in the large and small intestine by qPCR and immunofluorescence, and 32 of the subjects enrolled in the study resulted positive for viral RNA signal in their GIT 7 months after viral SARS-CoV-2 acute infection. Viral nucleocapsid (N) protein was detected in the gut epithelium and CD8^+^ cells from 24 of the 46 patients, even though no replicating SARS-CoV-2 could be recovered from the biopsies [38]. Interestingly, only subjects manifesting PASC symptoms have viral RNA in their GIT [38]. Stein and colleagues found viral sgRNA up to 76 days in the kidney, lymph nodes from the thorax, lung parenchyma, and salivary glands samples and detected signals for the N gene up to 230 days in biopsies from the central nervous system (CNS), ocular tissue, sciatica nerve, myocardium and pericardium, lung, and lower airways [39]. Yao et al., studying the epithelial layer and lamina propria of the fungiform papilla in patients with PASC experiencing taste deficits, were able to identify by RNAscope genomic positive-strand RNA of SARS-CoV-2 up to 40 weeks from acute infection from two patients and a significant IL-1β signal in infected papillae [40].

Rong et al. analyzed the localization of the S protein in mice after SARS-CoV-2 infection and identified that it accumulates in the skull bone marrow, leading to a hyperinflammatory state driven by neutrophil activation [41]. Interestingly, they also demonstrated that in a pool of 34 patients who died from non-COVID-related causes during the pandemic, 10 of them had S protein in their skulls, suggesting the ability of this protein to persist in this anatomical district [41]. Hout and colleagues infected a pool of macaques with Wuhan and Omicron variants of SARS-CoV-2 and detected, 221 days after infection, the persistence of viral antigens and nucleic acid in macrophages from Bronchial Alveolar Lavages (BAL), as well as replication-competent virus [42]. Furthermore, they observed that the spreading of SARS-CoV-2 from these cells was likely via a cell-to-cell mechanism, possibly avoiding the detection from other immune cells [42]. Finally, they reported that the peptide V_3–11_ generated by the signal peptide peptidase (SPP) for MHC exposure binds the MHC-E and inhibits the NK cell degranulation activities [42]. Patterson and colleagues showed that, after up to 15 months from the infection, the S1 subunit of the S protein of SARS-CoV-2 could persist in non-classical monocytes [43]. It is not entirely clear if and how these cells became infected, but it is possible that infection occurred during the early phase of development when monocytes express ACE2 and TMPRSS2 [43] or by antibody-dependent phagocytosis (ADP) [44]. The infection of non-classical monocytes may be related to a reduction of classical and intermediate monocytes and an expansion of non-classical monocytes [43] observed in PASC cases. In conclusion, there is strong evidence that SARS-CoV-2 NA and antigens can persist long-term after acute infection. This residual signal is found in various sites, often after the viral signal is no longer detectable in the upper respiratory system used for screening.

### 2.2. Immune System-Based Evidence

#### 2.2.1. Persistent Activation of the Immune System

Several lines of evidence show that SARS-CoV-2 can influence and alter the immune response even months after recovery from acute infection. For example, patients with prolonged olfactory symptoms are characterized by a protracted inflammation in their olfactory epithelium [45]. It has been shown that in the olfactory bulb of hamsters and humans, after recovery from acute symptoms, there is persistent immune activation with overexpression of genes involved in the complement system (see more below) and interferon response [46]. The same study shows that in the olfactory epithelium in both hamsters and humans, there is an enrichment of gene sets specific for T-cell activation (chemotactic genes and T-cell-associated genes) [46]. Another group, using an immune cell phenotyping, observed that, both at 3 months and 8 months after recovery from acute SARS-CoV-2 infection, there is chronic activation of CD8+ T-cells, expansion of plasmacytoid dendritic cells (pDC) and monocytes, accompanied by the absence of naïve T- and B-cells [47]. SARS-CoV-2 exhibited persistent effects on immune cells even months after recovery from acute infection, with a persistent conversion of naïve T-cells to the activated state, possibly as a result of persistent antigen presentation [47].

A clinical study of 17 children suffering from PASC showed SARS-CoV-2-specific CD4^+^ and CD8^+^ T-cell responses characterized by low antigen affinity [48]. The data suggested that the expansion of sub-optimal memory cells could prevent the development of a high-affinity T-cell response [48]. Similarly, a study in adult patients reported that higher numbers of CD4^+^ and CD8^+^ T-cells (expressing TNF-alpha and interferon-gamma) specific for SARS-CoV-2 N, membrane, (M), and S proteins were observed in PASC patients as compared to patients without PASC [49]. Visvabharathy and colleagues found that PASC patients suffering from neurological symptoms are enriched in CD4^+^ T-cells specific for N protein compared to individuals without PASC with a history of SARS-CoV-2 infection, coupled with impaired N-specific CD8^+^ memory responses [50]. This study is consistent with an omics analysis that compared 27 subjects with long-term symptoms of SARS-CoV-2 infection and 16 subjects who fully recovered from acute infection. The data showed that SARS-Cov-2 specific CD8^+^ T-cells from individuals with long-term symptoms frequently expressed markers of immune exhaustion PD1 and CTLA4, possibly due to chronic antigenic stimulation [51]. Another study performed on PASC patients reported expansion of CD8^+^ T-cells. However, these cells were characterized by slightly lower response against S protein and higher levels of PD-1, granzyme, perforin, and the transcriptional factor eomesodermin (EOMES) compared to patients without PASC. The phenotype of CD8+ cells was consistent with an activated state, suggesting that chronic activation is due to viral persistence of SARS-CoV-2 in GIT [52]. Interestingly, studies mentioned above detected the presence of N protein several months after acute SARS-CoV-2 infection in epithelial and CD8^+^ intestinal cells [38] and of the SARS-CoV-2 N gene in the CNS [39], possibly impairing the CD8^+^ response and the complete removal of viral antigen from the body.

As mentioned above, even innate immunity pathways are activated in PASC. For example, Cervia-Hasler and colleagues found that patients who eventually developed PASC displayed higher activity of the complement system during acute SARS-CoV-2 infection, and this activation was still detected during PASC, unlike in recovered patients [53]. Furthermore, complement activation was associated with a hemolytic process and the formation of aggregates between monocytes and platelets [53]. These findings suggest that chronic inflammation could lead to a heightened immune response in subjects affected by PASC symptoms.

#### 2.2.2. Effects of Vaccination on PASC

If PASC is caused by the persistence of SARS-CoV-2 or viral components, it is reasonable to infer that a vaccine, capable of inducing a protective response, should stimulate the immune system to eliminate residual antigens, resulting in prevention, or at least improvement of PASC symptoms, facilitating full recovery. While studies and case reports performed early in the pandemic, show only marginal benefits of vaccination on PASC symptoms [54,55], a large retrospective cohort study performed on more than 1.1 million patients infected with SARS-CoV-2 revealed that patients who received complete vaccination regimens with or without a booster dose were protected from clinical sequelae up to one year after infection as compared to unvaccinated individuals [56]. The level of protection was graded, being highest in individuals who had received one booster dose and lower in unboosted subjects [56]. Similarly, a multi-country, staggered cohort study that evaluated long-term symptoms 90 and 365 days after SARS-CoV-2 PCR positivity in more than 10 million vaccinees revealed a protective effect against long-term symptoms compared to unvaccinated individuals [57]. Also, a population-based cohort study performed on more than half a million COVID-19 patients in Sweden reported efficacy in reducing PASC symptoms of 21% for one dose of vaccine, 59% with two doses, and 73% with three doses [58]. An analysis of the Office of National Statistics (ONS) of the UK government showed a 13% reduction in the likelihood of PASC symptoms after the first dose of vaccine, followed by another 9% reduction after the second dose, with statistical evidence of a sustained improved of the quality of life [59]. A study on the effects of vaccination on PASC was performed on 812 individuals with a history of SARS-CoV-2 infection, with 72.4% of participants affected by PASC symptoms. In that study, after vaccination, 57.9% of the participants reported an improvement in PASC symptoms; another 17.9% experienced a temporary deterioration of symptoms that quickly resolved, suggesting that they were side effects of vaccination and not an aggravation of the disease [60]. While quantitative and qualitative differences were noted in the profile of activity of each vaccine approach in preventing PASC symptoms, all of the vaccines had some efficacy in lowering long-term symptoms [60]. Another study addressing the impact of the vaccine on PASC in 6729 participants indicated that the odds of developing PASC symptoms were reduced by 12.8% after the first vaccine dose, followed by another 8.8% after the second dose and a further 0.8% for every following week for the studied time [61]. Finally, a meta-analysis of 32 studies on more than 700,000 individuals revealed efficacies of 36.9 and 68.7% for two and three doses of vaccine, respectively [62]. Therefore, there is a preponderance of evidence for a protective role against PASC for SARS-CoV-2 vaccines.

#### 2.2.3. Prolonged Evolution of Antibodies

**Figure 2:**
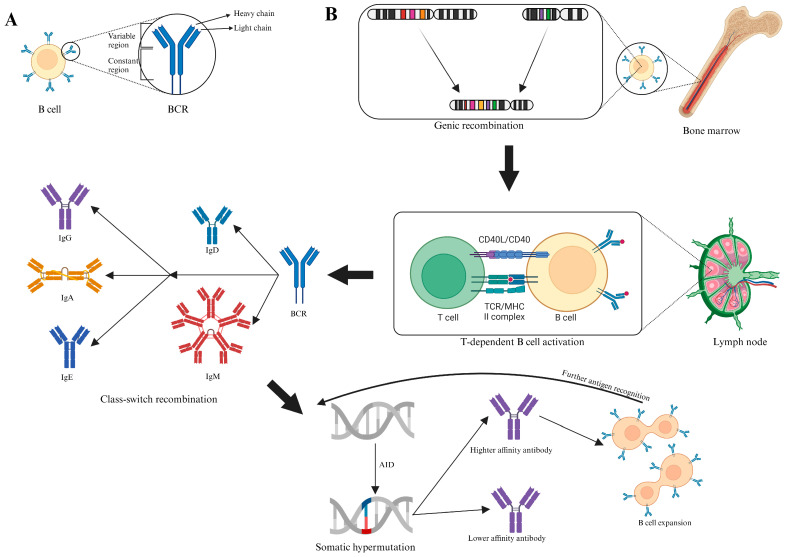
(**A**) Schematic representation of B cells, antigen receptors, and antibodies. (**B**) Schematic representation of B cell development and maturation. B cells originally develop in the bone marrow, where through gene recombination, they develop a specific antigen receptor on its membrane (B cell receptor, BCR). Subsequently, B cells migrate to a secondary lymphoid organ where they can become activated upon binding their cognate antigen, secreting IgM. B cells can internalize and process the antigen, presenting it in the context of Class II MHC to CD4 T-cells. Upon recognition of their cognate antigen, it can stimulate B cells (T-dependent B cell activation), inducing the class–switch recombination process, first leading to the switch of the antibody isotype from IgM or IgD to IgE, IgA, or IgG. Further stimulation of B cells from the cognate antigen induces the somatic hypermutation process, leading to the development of antibodies with higher affinity to the specific antigen. Created with BioRender.com.

The B cell receptor (BCR) is expressed on the surface of B cells, where it can recognize foreign antigens (Figure 2A) [63]. After the maturation and activation of B cells, the BCR is secreted outside the cells in the form of an antibody (Ab) (Figure 2B) [63]. After this process, B cells migrate from the bone marrow to the secondary lymphoid organs to bind the cognate antigen [63]. Here, by a T-dependent immune response, the B cell can be activated and undergo two main processes: class–switch recombination (CSR) and somatic hypermutation (SHM) [63]. The CSR process leads to a switch in the constant region of the BCR from IgD or IgM to the other types of antibody: IgG, IgA, or IgE [63]. Furthermore, with prolonged activation by the cognate antigen in the germinal centers (GC), SHM introduces new mutations in the V gene, changing the variable region of the final antibody and possibly potentiating the affinity between the antibody and its respective antigen [63]. If the newly developed antibody is more affine to the antigen, this cell is positively selected and expanded. Otherwise, it goes under negative selection; thanks to this process, antibodies with higher affinity for the antigen are developed after every event of antigen recognition [63].

Wu and colleagues, while isolating antibodies and B cells from a blood sample obtained from a convalescent patient in April 2020, before the development of SARS-CoV-2 variants, observed that some B cells produced an antibody capable of neutralizing a wide spectrum of variants, including omicron, likely because of SHM, highlighting the importance of this process in evolving improved antibodies [64]. Gaebler and colleagues studied the evolution of memory B cells after 1.3 and 6.2 months from the infection [65]. They reported that after the infection, there is a rapid turnover of memory B cells and a continuous SHM-driven evolving of antibodies, even if these patients were not reinfected, generally leading to better neutralizing antibodies [65]. Furthermore, they found that individuals suffering from PASC symptoms had significantly higher levels of anti-RBD and anti-N IgG than individuals without persistent symptoms [65]. Similar data on higher levels of SARS-CoV-2 antibodies in PASC patients as compared to non-PASC controls were reported by other groups [51,66]. The persistent evolution of antibodies could be caused by antigens trapped inside the immune complexes in the lymph node or due to virus persistence within the body. To identify the cause behind this prolonged SHM, this group analyzed GIT from 14 subjects after 4 months of SARS-CoV-2 infection, and 5 resulted positive to immunostaining against N protein, 3 samples were able to produce SARS-CoV-2 PCR amplicons, and in 2 samples viral RNA was identified by in situ hybridization. These findings suggest that the persistent evolution of antibodies is related to the persistence of viral antigens [65].

Manthiram’s group studied the adaptive immune response in tonsils and adenoids sampled from children 102 days after initial SARS-CoV-2 infection [67]. They found a robust humoral response against SARS-CoV-2, and analysis of the BCR sequence of B cells specific for the subunit 1 of the SARS-CoV-2 S protein (S1^+^ B cells) showed that these cells were primarily IgG1 and IgA1 with a high frequency of SHM with a lower clonal diversity compared to S1^-^ B cells indicating that these cells derived from the expansion of one GC [67]. Upon analyzing the CD4^+^ cells, they identified a combination of markers associated with IFN-γ and Th1 cytokine production, suggesting a Th1-driven immune response [67]. Furthermore, they detected an expansion of CD4^+^ cells expressing CD57, a marker of cell senescence and usually associated with chronic infection [67,68]. Enrichment of CD8^+^ tissue-resident memory T cells (CD8^+^T_RM_) cells was detected in the tonsils and adenoids of children previously infected [67]. To identify if this persistent immune response involving T and B memory cells and expansion of GC was caused by the persistence of SARS-CoV-2, digital droplet PCR (ddPCR) was performed, and 7 out of 9 adenoid and 15 out of 22 tonsil samples resulted positive to viral nucleocapsid RNA [67]. Finally, Sokal and colleagues performed a longitudinal analysis up to 6 months after SARS-CoV-2 infection of B cells, finding a progressive mutation in the VH sequence between the third and sixth month after acute infection, showing a continuous SHM and antibody evolution over time [69]. The continuing evolution of antibody response is consistent with the observed persistence of viral nucleic acids and antigens, thus reinforcing the concept that virus components persist long after the initial symptoms of COVID-19.

## 3. Discussion

COVID-19 has created a unique situation: the entire world population, previously immunologically naïve to SARS-CoV-2, has been exposed to infection. Even if vaccination ameliorates both acute and post-acute symptoms, large segments of the population are not vaccinated. Therefore, even relatively infrequent post-acute complications of RNA virus infection result in a large number of subjects affected. It is estimated that more than 65 million individuals are currently suffering from PASC while the mechanisms behind these symptoms are not known, and the hypothesis proposed spans from tissue damage, a persisting reservoir of SARS-CoV-2, an immune dysregulation, reactivation of chronic pathogens, an alteration of the microbiota, autoimmunity, and vascular issues [10,70]. In reality, post-acute sequelae are not unique to SARS-CoV-2, as similar findings had been reported for the previous SARS and MERS outbreaks [71,72,73], but fortunately, neither situation resulted in a number of cases even comparable to COVID-19.

The mechanism behind the persistence of RNA viruses after the acute phase remains unknown, but SARS-CoV-2 is not the only RNA virus able to persist [74]. For example, *Ebolavirus* can persist in anatomic sides protected from the immune system, such as the brain and testes, enabling sexual transmission of the virus weeks after the recovery of the patients [74]. Interestingly, post-Ebola syndrome has also been reported [75,76,77]. The variety and severity of symptoms may be due to both unique characteristics of SARS-CoV-2 and its interaction with each infected individual, as it was recently reported [78], but also to the extremely high number of cases in a previously immunologically naïve population. Thus, countless interactions could eventuate in a plethora of mechanisms and symptoms, requiring the formulation of a detailed framework to interpret the complexity of PASC [12,13]. It is possible that trying to group all of these mechanisms and outcomes into a single denomination, such as post-COVID-19, PASC, long-COVID, or other definitions, may have done a disservice to the field, and it may just be more accurate to refer to every single symptom as such. The same consideration applies to the identification of a common set of biomarkers for all of these disparate conditions.

One rather puzzling aspect of PASC is that while most of the evidence of persistence of SARS-CoV-2 in the body points to the GIT as a possible antigen reservoir, most of the symptoms, such as fatigue or attention disorder, are linked to CNS. The persistence of SARS-CoV-2 in GIT could perhaps induce a local immune response, shaping the local microbiota for prolonged periods. This suggests a possible role of the gut–brain axis (GBA) in this pathology, and gut and oral dysbiosis have been proposed to cause the insurgence of PASC [79,80]. The GBA consists of a complex and bidirectional interplay between the CNS and gut microbiota by vagal nerve, hormones, and immune system [81]. A healthy and balanced microbiota is responsible for maintaining the integrity of the epithelial barrier, while the reduction of butyrate-producing and mucin-degrading bacteria increases the gut epithelium permeability, allowing LPS and bacterial antigens to pass through the epithelium [81]. In the end, this leak triggers a strong immune response affecting even the blood–brain barrier (BBB), stimulating neuroinflammation since LPS can directly affect the BBB integrity [81]. Furthermore, the microbiota is responsible for metabolizing numerous dietary components in short-chain fatty acids (SCFA) (mainly acetate, butyrate, and propionate acids) and trimethylamine N-oxide (TMAO) [82].

Bacteria of microbiota can also stimulate a local immune response or metabolize the tryptophan, limiting its use as a precursor of indole, melatonin, and serotonin, low levels of which have been linked to neurological disorders [83]. Finally, microbiota can directly synthesize neurotransmitters [83]. The role of gut microbiota in neuronal health is highlighted by the association between gut dysbiosis and multiple neurological disorders, such as Alzheimer’s disease, Parkinson’s disease, multiple sclerosis, epilepsy, and sleep disorders [49]. Accordingly, Wong et al. reported that serotonin levels are reduced during the acute and post-acute sequelae of a viral infection, SARS-CoV-2 included [84]. The observed reduction of serotonin is caused by a reduction of the intestinal absorption of tryptophan, and this negatively impacts the activity of the hippocampus and cognitive function through the vagal nerve [84]. Finally, a chronic viral infection can also lead to these effects on serotonin levels, suggesting that PASC could be caused by unresolved inflammation due to the persistence of some viral component [84]. Relevant to the role of microbiota in brain physiology, inflammatory bowel disease is associated with an alteration of microbiota, and half of the patients manifest fatigue as a symptom [85,86]. Both SCFA and TMAO are involved in cardiovascular health. For example, sodium butyrate, a butyrate derivative, reduces cerebral infarction and apoptosis in ischemic stroke [82]. SCFA promotes peripheral CD4+ T cell activation and increases the anti-inflammatory cytokine IL-22 [82]. TMAO manifests opposite effects given that it can directly activate platelets, augmenting the likelihood of thrombosis and stroke as well as increasing the expression of pro-inflammatory cytokines, such as TNF-α, IL-6, and C reactive protein [82]. Symptoms-specific alteration in gut microbiota 6 months after SARS-CoV-2 infection has been described [87]. Specifically, patients with prolonged respiratory symptoms (cough, shortness of breath) were found to have an expansion of opportunistic pathogens (*Streptococcus* species including *S. anginosus*, *S. vestibularis*, *S. gordonii,* and *Clostridium disporicum*). An enrichment of nosocomial pathogens linked to opportunistic infection (*Clostridium innocuum* and *Actinomyces naeslundii*) was found to be associated with neuropathological symptoms and fatigue [87]. In addition, the relative abundance of bacterial species beneficial for the regulation of immune response and for the production of butyrate (*Bifidobacterium pseudocatenulatum*, *Faecalibacterium prausnitzii*, *Roseburia hominis, and R. inulinivorans*) were inversely correlated with PASC symptoms [87]. Another analysis of the gut microbiome in PASC found a correlation between IL-6 and CRP levels and microbiome composition, with lower values of the ratio of abundance of *F. prausnitzii* to Bacteroides species in PASC patients compared to patients without PASC [88]. Kusakabe et al. reported an alteration in the mycobiota of the GIT in patients with severe COVID-19 caused by *Candida albicans* overgrowth and suggested that this contributes to PASC [89].

## 4. Conclusions

**Figure 3:**
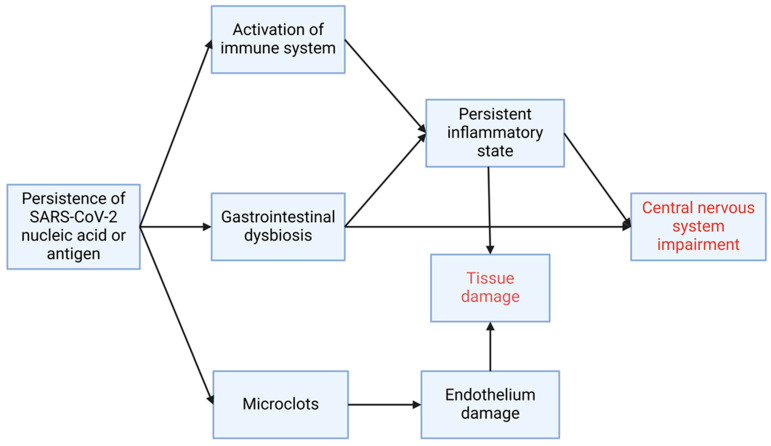
The persistence of SARS-CoV-2 nucleic acid or antigen can stimulate and activate the immune system, leading to a persistent inflammatory state, causing tissue damage, and possibly impairing the function of the CNS. The persistence of antigens or NA in the gastrointestinal system can cause a dysbiosis that can further enhance the inflammatory state or the gut–brain axis (GBA) has an impact on the CNS. Finally, viral antigens can stimulate the formation of microclots, causing damage to endothelium and tissue. Created with BioRender.com.

The persistence of SARS-CoV-2 or its component could be a driver of most of the mechanisms behind PASC symptoms. Firstly, the continuous exposure of viral antigens keeps the immune system constantly active, causing persistent tissue inflammation (Figure 3). This exacerbated immune response, coupled with coagulation abnormalities (see below), could directly damage the tissue or, in the long period, lead to the development of auto-immune disease. If the persistence of viral antigens happens in the gut, as many reports cited above suggest, it could affect, together with immune system activation, the local microbiota (Figure 3). Secondly, the persistence of the spike protein (including in non-classical monocytes that in PASC are expanded in various compartments, including the endothelium [90,91]) could induce the formation of fibrinaloids causing local microclotting (Figure 3, and Section 1). More clinical and basic research is needed to clarify the ability of SARS-CoV-2 to remain for a prolonged period in the human body, the possible role of gut microbiota in PASC, and develop better therapies enabling the full recovery from this disease. While the body of studies described above is impressive, many questions remain, not just, as we highlighted, on the pathogenic mechanism of PASC; it is still unclear why women, who are less likely to develop severe acute symptoms than men, have instead an increased risk of developing PASC [92]. Besides the potential role of estrogen in accelerating fibrinaloids formation (see above), reports have started dissecting sex-specific differences in the immune response to SARS-CoV-2 [51,93]. Further, studies assessing the impact of PASC in children report widely different results in the odds of having long-term symptoms, varying from relative risk similar to that of adults to marginal risk [94,95]. The reason for these discrepancies may be ineffective clinical follow-up, underreporting, and lack of informative disease biomarkers and disease definition. However, it is critical to readdress the impact of PASC on both women and children and identify strategies to treat its symptoms.

## Data Availability

This manuscript reviews data published in the literature. Information on data availability for each article cited can be found in the source article.
